# Novel Cellulose Films Obtained by the Combination of High-Pressure and Cellulase Treatments

**DOI:** 10.3390/ma19163552

**Published:** 2026-08-21

**Authors:** Gonçalo Coelho, Renata A. Amaral, Daniela M. Santos, Jorge A. Saraiva

**Affiliations:** Associated Laboratory for Green Chemistry-Network of Chemistry and Technology (LAQV-REQUIMTE), Department of Chemistry, University of Aveiro, 3810-193 Aveiro, Portugal; goncalocoelho@ua.pt (G.C.); renata.amaral@ua.pt (R.A.A.); danielams@ua.pt (D.M.S.)

**Keywords:** films, cellulose, cellulase, high pressure processing, enzymes

## Abstract

This research study focused on the use of technologies such as high-pressure processing (400 MPa for 15 min, HPP) and enzymatic hydrolysis (with cellulase) to process eucalyptus pulp, subsequently the pulp submitted to carboxymethylation to produce cellulose films and investigate their properties. The results revealed that the combination of HPP and cellulase resulted in the production of partially transparent and smoother films. As for mechanical properties, this combination resulted in a 2.7-fold increase in the tensile strength (TS) value, a 35.5-fold increase in the elongation at break (EAB) value, and a 1.4-fold increase in the moisture content of the films. The crystallinity index (CrI) was also increased by HPP and enzymes, resulting in a 6.6-fold increase, compared to the control film. On the other hand, the combined use of HPP and enzymatic hydrolysis resulted in similar contact angle (CA) values on both sides of the films, but in the bottom side, when compared to the control film, a 2.1-fold decrease was observed. Furthermore, the water vapor permeability (WVP) of the films increased 1.8-fold. Finally, the thermal resistance of the films was slightly reduced when either HPP, enzymatic or both treatments were used on the cellulose pulp. In general, this work showed a new potential way to produce cellulose films with novel and potentially tailor-made properties.

## 1. Introduction

Nowadays, synthetic materials like plastics have become indispensable in our daily lives. The primary reason behind their widespread usage is their ease of handling, affordability, and commendable mechanical, and barrier characteristics. These attributes have fueled their rapid proliferation and extensive utilization, particularly in the domains of food packaging and healthcare. Nevertheless, the growing apprehension among consumers regarding the environment, the depletion of fossil fuels, and the adverse ecological consequences associated with these materials lead to a need for their replacement with alternative resource-based materials, such as natural polysaccharides [1].

Synthetic materials such as plastics have become indispensable because of their low cost, ease of processing, low density, and favorable mechanical and barrier properties. These characteristics support their extensive use in economically important sectors, particularly packaging and healthcare. The global economic relevance of plastics is quite considerable, for example, in 2019, plastics production represented approximately 1.3% of the global economy, while the monetary value of plastics used across economic activities was estimated at USD 4.9 trillion [2]. In the same year, global plastics use reached approximately 460 million tonnes, with packaging, construction, and transportation collectively accounting for more than 60% of the total demand [3]. However, this extensive use is associated with considerable environmental impacts. Approximately 353 million tonnes of plastic waste were generated globally in 2019, of which only 9% were ultimately recycled [2]. These economic and environmental considerations reinforce the need to develop materials derived from renewable resources, including natural polysaccharides, as potential alternatives to conventional fossil-based plastics [1].

Among these natural polysaccharides, cellulose stands out as the most abundant one globally [4,5]. Cellulose possesses remarkable characteristics such as renewability, non-toxicity, biodegradability, high thermal stability [6], and cost-effectiveness [7], which contributes to its significant role as a valuable biopolymer in various commercial applications [8]. Notably, cellulose has demonstrated great potential in film production [5], having increasingly been studied in the production of edible films to improve the properties and increase the shelf-life of food products [8].

Cellulose films have garnered significant research attention due to their remarkable characteristics, including sustainability, biocompatibility, mechanical and barrier properties, and affordability [9]. Nevertheless, these films still possess certain limitations, like the insolubility of cellulose in water that restricts the potential for new film advancements and further applications of cellulose [10]. Therefore, it is crucial to persist in exploring novel technologies that can modify the structure and properties of cellulose, thereby addressing the issue of insolubility and other limitations of cellulose films.

High-pressure processing (HPP) is a novel technology that shows great promise as a non-thermal processing method. HPP involves subjecting products to a range of pressures between 100 and 600 MPa, typically at room temperature [11]. The application of this technology is based on two fundamental principles. The first principle, known as Le Chatelier’s principle, states that an increase in pressure leads to an enhancement of all reactions and changes that favor the decrease in pressure. The second principle, known as the isostatic principle, asserts that pressure is uniformly applied to the product, regardless of its shape/size. Unlike conventional thermal technologies, HPP primarily affects mainly non-covalent bonds, particularly hydrogen bonds [12] and has minimal impact on covalent bonds. This characteristic makes HPP suitable for modifying the structure and properties of macromolecules such as starch or cellulose [13].

HPP may also be combined with enzymatic treatments to increase the accessibility and controlled modification of cellulosic fibers. Figueiredo et al. [13] showed that the treatment of eucalyptus pulp at 400 MPa promoted structural rearrangements of cellulose, increased the amount of strongly bound water, and improved the accessibility of amorphous domains. These pressure-induced changes may facilitate enzyme penetration and action within the fiber wall.

Ferreira et al. [14] subsequently demonstrated that pretreatment of eucalyptus bleached kraft pulp at 300–400 MPa increased both the rate and degree of subsequent cellulose hydrolysis by cellulase, resulting in approximately 1.5- to 1.9-fold higher reducing-sugar formation. The authors attributed this effect to increased cellulose accessibility and proposed the approach primarily for improving the production of fermentable sugars and other cellulose-derived chemicals.

Using the same type of eucalyptus kraft pulp, Oliveira et al. [15] investigated HPP pretreatments followed by xylanase hydrolysis, and observed that pretreatments at 300–400 MPa increased the initial rate of xylan hydrolysis by approximately 5- to 10-fold and promoted disaggregation of hydrated cellulose fibrils. These results demonstrated that pressure and treatment time could be used to regulate the extent of enzymatic modification within cellulosic fibers. Salgueiro et al. [16] later applied HPP followed by xylanase treatment to recycled hardwood pulp. The combined treatment increased fiber accessibility and improved the mechanical properties of the resulting paper sheets, with increases of up to approximately 30% in some strength parameters.

The aforementioned studies demonstrated that HPP can enhance enzymatic accessibility and promote the controlled modification of cellulosic fibers; nevertheless, previous studies have mainly focused on enzymatic hydrolysis kinetics, fermentable-sugar production, pulp modification, or papermaking properties. The influence of sequential HPP and cellulase treatment on the subsequent carboxymethylation, film-forming capacity, and properties of cellulose films remains scarcely reported in the literature.

Cellulose also has the potential to undergo chemical modifications, including alkalinization and carboxymethylation, to modify its properties and transform its structure [17]. Carboxymethylation, which is a cost-effective and relatively straightforward chemical treatment [18], involves a chemical reaction between cellulose and monochloroacetic acid in the presence of sodium hydroxide [17]. This reaction primarily leads to surface modifications, resulting in enhanced solubility of cellulose through the introduction of carboxymethylated groups. Consequently, this process overcomes the significant challenge of cellulose solubility and facilitates its utilization in various applications, such as film production [18]. Therefore, the present exploratory study investigates a distinct sequential strategy involving HPP and endocellulase treatment of eucalyptus bleached kraft pulp, applied individually or in combination, followed by carboxymethylation and film formation. To the best of our knowledge, the combined influence of these pretreatments on the physicochemical, mechanical, barrier, optical, morphological, thermal, and structural properties of the resulting carboxymethylated cellulose films has not previously been systematically evaluated. The study was designed to provide first insights into whether HPP and cellulase pretreatments can be used to obtain films with distinct and potentially tailorable properties. The films could be expected to demonstrate variations in visual appearance, moisture content, contact angle, water vapor barrier performance, mechanical properties, color parameters, surface morphology, thermal resistance, crystallinity index, and chemical composition (as analyzed by Fourier Transform Infrared, FTIR).

## 2. Materials and Methods

### 2.1. Materials

**Figure 1:**
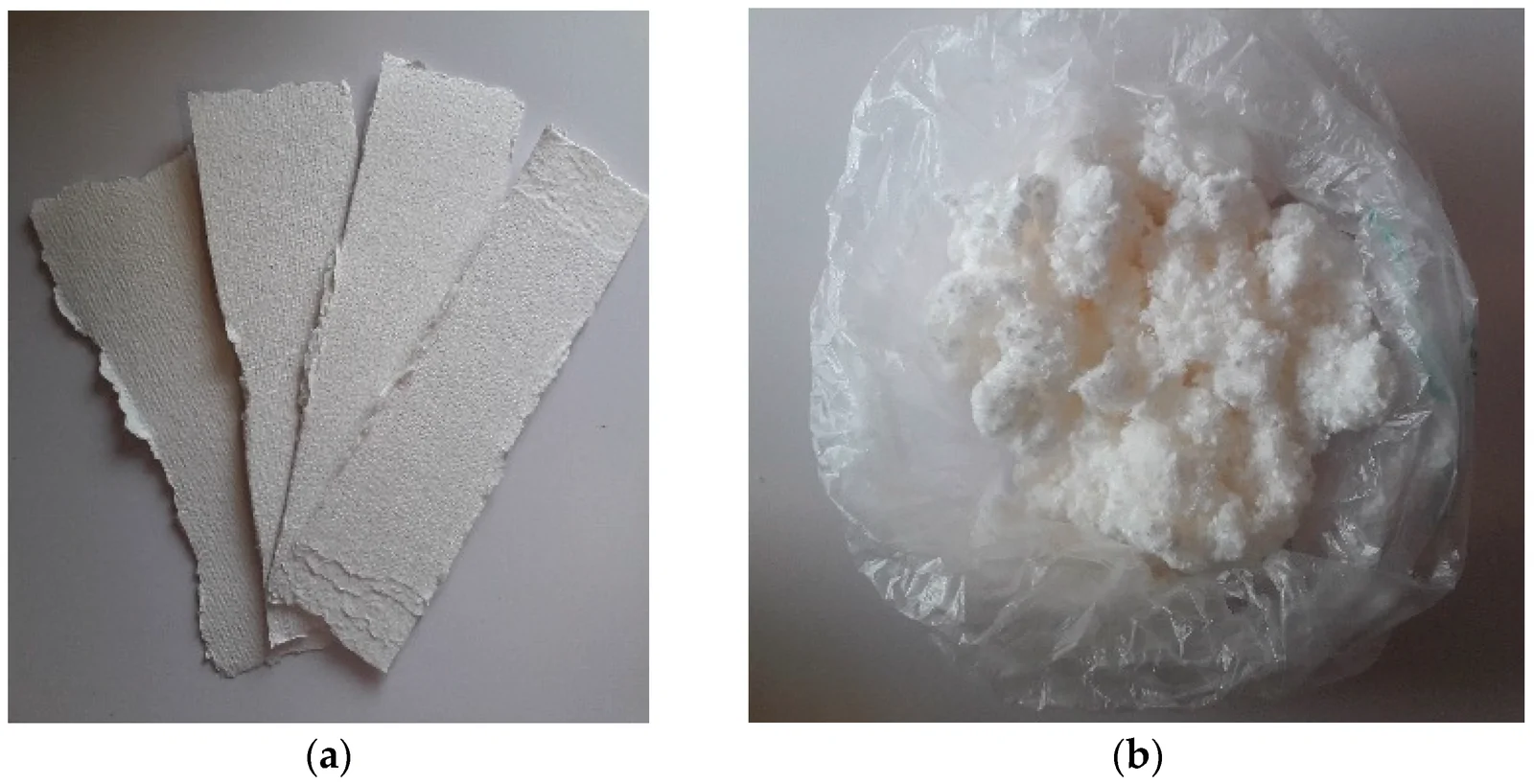
Eucalyptus bleached kraft pulp. (**a**) In its raw state. (**b**) After maceration.

The organic solvents used during this work were absolute ethanol (≥99.8%, CAS 64-17-5) purchased from Fisher Scientific and glacial acetic acid (≥99%, CAS 64-19-7) purchased from Sigma-Aldrich (St. Louis, MO, USA). The reagents used include sodium hydroxide (CAS 1310-73-2) purchased from Merck (Darmstadt, Germany) and sodium chloroacetate (CAS 3926-62-3) purchased from Sigma-Aldrich. All reagents were commercially purchased and used without purification. The source of cellulose used was a eucalyptus (*Eucalyptus globulus*) bleached kraft pulp kindly supplied by a Portuguese pulp mill. The pulp was received in its raw dry state (Figure 1).

The molecular weight and composition (cellulose, hemicellulose, lignin) of this pulp was supplied by the provider and is present in Table 1.

The enzyme used consisted of an endocellulase enzyme solution (NS 280430) kindly provided by Novozymes (Bagsværd, Denmark). The carboxymethylated cellulose used to produce the films was produced in our laboratory (as described in the procedure below), as such it was not characterized in terms of its molecular weight or composition.

### 2.2. Cellulose Eucalyptus Pulp Treatment

**Figure 2:**
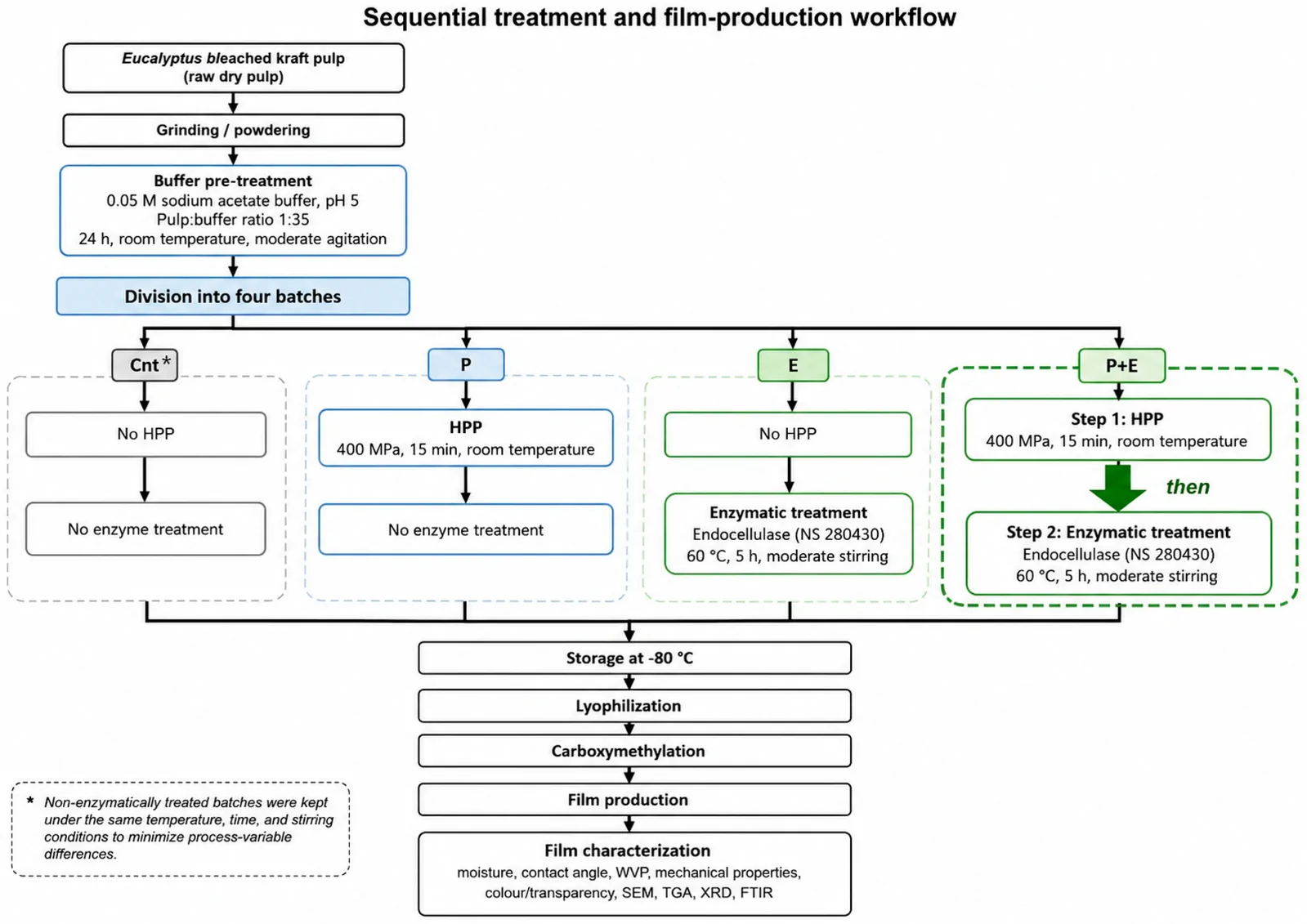
Schematic representation of the sequential treatment applied to eucalyptus pulp, and film production workflow. After buffer pre-treatment, the pulp was divided into four batches: Cnt (no HPP, no enzyme), P (HPP only), E (enzymatic treatment only), and P+E (HPP followed by enzymatic treatment). After the respective treatments, all samples were frozen, lyophilized, carboxymethylated, and used for film production.

Two main treatments, HPP and enzymatic hydrolysis with cellulase, were performed on the pulp, to study their effects on cellulose and how it would influence the properties of the final produced films. The following sequence of work was performed (Figure 2).

Initially, before any treatment, the eucalyptus pulp was ground into “powder”. After that, by adapting the work of Oliveira et al. (2012) [15], the pulp went through a pre-treatment step with a sodium acetate buffer (0.05 M) pH 5, in a ratio of 1:35 (pulp:buffer). The pulp powder was added to the buffer and swollen, under moderate agitation and at room temperature, for 24 h. Once this was done, four batches of pulp pre-treated with the buffer were processed by four different treatments: Cnt—The first batch was a control, where it was neither treated by HPP nor enzymes; E—The second batch was only submitted to an enzymatic treatment; P—The third batch was treated only by HPP; P+E—The fourth and last batch was treated by HPP and enzymes. Once the treatments were concluded, the final samples were stored at −80 °C for posterior lyophilization, which also allowed the stopping of the enzyme activity. Finally, since these treatment steps were not enough to solubilize cellulose and produce the films, a carboxymethylation step was adapted from Qi et al. (2021) [19], and performed to enhance the solubility of cellulose and produce the desired films.

#### 2.2.1. HPP Pre-Treatment

About 500 mL of pulp suspension (batches P+E and P), containing around 14.29 g of dry pulp powder (ratio of 1:35, 1 g of dry pulp powder to 35 mL of buffer) were heat-closed into polyamide/polyethylene (PA/PE) bags. The PA/PE bags were then subjected to 400 MPa for 15 min at room temperature. The treatment was performed using industrial-scale equipment with a 55 L capacity (model 55, Hiperbaric, Burgos, Spain) with water used as the pressurization fluid.

#### 2.2.2. Enzymatic Treatment

Previous studies with different enzyme concentrations did not reveal any significant changes on the cellulose pulp, therefore an intermediate enzyme concentration of 45.27 µL of the endocellulase enzyme solution was added to 500 mL of pulp suspension (batches E and P+E), containing around 14.29 g of dry pulp powder. The suspension was poured into an Erlenmeyer and placed in an oven, pre-heated at 60 °C, for 5 h with moderate stirring. After that, the suspension was divided into 50 mL plastic cups and stored at −80 °C until lyophilization, to stop the enzyme activity. Although not all batches went through the enzymatic treatment, they were all placed in the reactor for 5 h with stirring, to simulate the enzymatic treatment and reduce the number of variables throughout the process.

#### 2.2.3. Cellulose Carboxymethylation

As mentioned before, to enhance the cellulose solubility, a carboxymethylation process adapted from Qi et al. (2021) [19] was performed. After lyophilization, 10 g of the dried resultant pulp powder and 160 mL of absolute ethanol were added to a 500 mL Erlenmeyer, to the resultant suspension were also added 40 mL of an aqueous NaOH solution (15 *w*/*w*%). This mixture was heated to 30 °C for 60 min with constant stirring. After this alkalinization step, 12 g of sodium chloroacetate was added to the mixture and heated up to 65 °C for 2.5 h at moderate stirring. Thereafter, the pH of the resultant mixture was adjusted to 8, using acetic acid. Finally, with the help of a centrifuge (Hearus Biofuge Stratos Centrifuge from Thermo Electron Corporation, Waltham, MA, USA), the mixture was centrifugated five times with absolute ethanol to wash the pellets. The resultant pellets were dried overnight at 60 °C and after crushing the dried pellets, a thin white powder was obtained and stored in a desiccator with controlled humidity conditions. This procedure was repeated to all four different batches (Cnt, P, E, and P+E) with a slight difference in the P and Cnt batches. In these two batches, the powders obtained after lyophilization were thicker and by adding only 160 mL, the stirring process became difficult. Knowing that the ethanol volume was increased to 240 mL to make the solution more liquid to allow the stirring process.

### 2.3. Production of Films

The production of the cellulose films was also adapted from Qi et al. (2021) [19], however, small changes were performed. This way, within a 500 mL Erlenmeyer, 6.0 g of carboxymethylated cellulose powder was added to 300 mL of an ultrapure water and ethanol solution mixture (*v*/*v* = 8/2). This mixture was then heated for 15 min at 90 °C, with moderate stirring. The obtained semi-transparent solution was degassed and filtered. Finally, the solution was cast into plexiglass plates and dried overnight at 40 °C in a ventilated oven. This procedure was repeated for every batch (Cnt, P, E, and P+E) to obtain four distinct types of films. The obtained cellulose films were then stored at controlled humidity conditions (approximately 53% RH, at room temperature) for future analysis.

### 2.4. Film Analysis

Before any analysis, the films were stored at controlled humidity conditions (≈53% RH, at room temperature) for at least 3 days to enable the stabilization of the material.

#### 2.4.1. Moisture

To measure the moisture and solubility of the films, three square replicates (2 × 2 cm) of each type of film (Cnt, P, E, and P+E) were cut and weighted (Initial Weight). After that, the squares were dried for 24 h in a ventilated oven at 105 °C. The moisture parameter was obtained using Equation (1).

(1) $$\%\;Humidity=\frac{Initial\;Weight-Dry\;Weight}{Initial\;Weight}\times 100$$

#### 2.4.2. Wettability and Water Vapor Permeability

Three rectangles of 10 × 1 cm from three different films of each type (Cnt, P, E, and P+E) were cut and divided in half to study both sides of the films and compare their wettability (WT). These rectangles were placed on microscope slides and the average contact angle was measured. The measurement was performed using a contact angle analyzer (OCA 25 Data Physics Corp., San Jose, CA, USA—SCA 20 3.16.1 software). The WT of the film rectangles was determined by accessing the angle formed by a 3 µL distilled water drop on the film surface (sessile drop). The equilibrium of the liquid surface was defined by Laplace-Young fitting and the contact angles were determined at least six times per sample, on both sides of the films.

To determine the water vapor permeability (WVP), three squares of 2.5 × 2.5 cm from each type of film (Cnt, P, E, and P+E) were cut. After that, 11 g of dry silica was weighed and placed inside the WVP plexiglass cups, the squares were placed on top of the capsules, and the rest of the WVP system was assembled. The capsules already assembled were weighed (initial weight) and stored at controlled humidity conditions (53% RH). For 12 h, the capsules were weighed from 30 to 30 min during the initial 2 h, and from 1 to 1 h until the rest of the time.

To determine the WVP of the films, the variation in the weight during the 12 h was plotted, and a linear regression was made. The values of the WVP were then determined by the following Equation (2).

(2) $$WVP\;(g/m\cdot s\cdot Pa)=C\times \frac{x}{A\times ∆P}$$

where x is the film thickness (m), A is the exposed area of the film through what the water vapor can be exchanged (m^2^), ΔP is the water vapor pressure differential across the film (calculated as 3169 Pa at 25 °C), and C is the slope of the weight gain of the WVP plastic cups (g), versus time (h).

#### 2.4.3. Mechanical Properties

To characterize the mechanical properties of the cellulose films, uniaxial stress tests were performed using a TA.HDi Texture Analyser (Stable Micro Systems^®^, Surrey, UK) at controlled humidity and temperature conditions (≈53% RH, 25 °C). For each type of film (Cnt, P, E, and P+E), at least 12 specimens were analyzed. These specimens had dimensions of 100 × 10 mm and each one had two sections that would not be exposed to tension and that would be used for the adhesion of the grips (25 × 10 mm for each claw). The films’ mechanical force was determined at a crosshead speed of 0.5 mm/s, and the stress tests were performed until the specimens ruptured. Measuring the tension with A/TG—Tensile Grips allowed the determination of the values of stiffness (Young’s Modulus), TS at breakage, and EAB.

Before performing the stress tests, the specimen’s thickness was also measured using a handheld digital micrometer (Digital Micrometer series 293 MDC Lite from Mitutoyo Corporation, Kawasaki, Japan) with an accuracy of 0.0001 mm. The thickness was measured at four different specimens.

The initial cross-sectional area of each specimen (A_0_, mm^2^) was calculated from its width and mean thickness according to Equation (3):

(3) $$A_{0}=w\times d$$

where (w) is the specimen width (mm) and (d) is the mean specimen thickness (mm), calculated from measurements performed at four different positions.

The engineering stress (σ, MPa) and engineering strain (ε, dimensionless) were calculated according to Equations (4) and (5), respectively:

(4) $$\sigma =\frac{F}{A_{0}}=\frac{F}{w\times d}$$

(5) $$\varepsilon =\frac{\left(L-L_{0}\right)}{L_{0}}$$

where F is the tensile force applied to the specimen (N), A_0_ is the initial cross-sectional area of the specimen (mm^2^), w is the specimen width (mm), d is the mean specimen thickness (mm), L_0_ is the initial distance between the grips (mm), and L is the distance between the grips during the test (mm).

The tensile strength at break (TS, MPa) was calculated according to Equation (6):

(6) $$TS=\frac{F_{\beta }}{A_{0}}=\frac{F_{\beta }}{w\times d}$$

where F_β_ is the tensile force at specimen rupture (N).

The elongation at break (EAB, %) was calculated according to Equation (7):

(7) $$EAB=\frac{\left(L_{\beta }-L_{0}\right)}{L_{0}}\times 100$$

where L_β_ is the distance between the grips at specimen rupture (mm).

Young’s modulus (YM, MPa) was calculated as the slope of the initial linear region of the engineering stress–strain curve, according to Equation (8):

(8) $$YM=\frac{{∆}_{\sigma }}{{∆}_{\varepsilon }}=\frac{\left(\sigma_{2}-\sigma_{1}\right)}{\left(\varepsilon_{2}-\varepsilon_{1}\right)}$$

where σ_1_ and σ_2_ are two engineering stress values within the initial linear region of the stress–strain curve, and ε_1_ and ε_2_ are the corresponding engineering strain values.

#### 2.4.4. Color Parameters and Transparency

To measure the color parameters (L*, a*, b*), a Konica Minolta CM-2300d colorimeter (Konica Minolta, Osaka, Japan) and the Spectra Magic NX software (version CM. S100W) were used. Three squares of 2 × 2 cm of the four types of films (Cnt, P, E, and P+E) were cut and used in three successive readings each.

The transparency was also measured and for that, the films were cut into a rectangular shape (12 × 8 cm) and placed inside the plate carrier of a Multiskan™ GO Microplate Spectrophotometer (Thermo Scientific™, Waltham, MA, USA). The absorbance was measured at 600 nm with the SkanIt software (version 6.1), always using the same position for the four types of films (the top side of the film facing the bottom of the plate carrier). To remove possible environmental interferences, a white measurement (with no film on the plate carrier) was performed and the absorbance obtained was subtracted from the values measured on the films. The transparency was then expressed in the form of transmittance at 600 nm, calculated through Equation (9).

(9) $$A=-\log_{10}T$$

where A is the absorbance at 600 nm, and T represents the transmittance at 600 nm.

#### 2.4.5. Surface Morphology

The surface morphology of the cellulose films was evaluated by scanning electron microscopy (SEM), where a scanning electron microscope (TMPlus 400 Hitachi, Tokyo, Japan) was used. Each type of film (Cnt, P, E, and P+E) was cut and trapped on an aluminum stub with carbon adhesive tape. The films were all evaluated on the top side with an acceleration voltage of 5 kV under vacuum conditions. No contrast agents were used to perform the surface morphology analyses. For each film, three images of three different sites of the film were recorded at two different magnifications, 300× and 1000×. The most representative photos of each film were used.

#### 2.4.6. Thermogravimetric Analysis

The TGA of the films was performed with the help of a simultaneous thermal analyzer STA 449 Jupiter (NETZSCH, Selb, Germany). Around 10 mg of each type of film (Cnt, P, E, and P+E) were weighed and analyzed using a heating rate of 10 °C/min from room temperature to 600 °C, under a nitrogen flow.

#### 2.4.7. X-Ray Diffraction

To obtain the X-ray diffraction (XRD) pattern of the films, one square (1 × 1 cm) of each type of film (Cnt, P, E, and P+E) was cut. The equipment used was a Rigaku SmartLabSe diffractometer (Rigaku, Tokyo, Japan) with a cobalt tub, graphite monochromator, and scintillation detector, used under the conditions of 40 kV and 30 mA. The film X-ray diffractograms were recorded on the SmartLab Studio II software 5.0 from 5° to 40° (2θ) with a scanning speed of 5 °/min. The crystallinity index (CrI) (%) of the films was calculated by Equation (10).

(10) $$CrI\;(\%)=\frac{A_{c}}{A_{T}}\times 100$$

Ac corresponds to the crystalline domain area, and AT corresponds to the total domain area.

#### 2.4.8. FTIR Analysis

To collect the FTIR spectra of the films, a Perkin-ElmerSpectrum BX FTIR spectrophotometer (Perkinelmer Inc., Waltham, MA, USA) equipped with a single horizontal Golden Gate ATR cell was used. The films were cut into squares of 1 × 1 cm and five replicates of each type of film (Cnt, P, E, and P+E) were analyzed at a range of 4000–400 cm^−1^, at a resolution of 4.0 cm, and averaged over 32 scans. The most representative spectra of each type of film were used.

### 2.5. Statistical Analysis

The statistical analysis of all data obtained throughout the work was done by one-way analysis of variance (ANOVA) using the Statistica software (version 10) (Statsoft, Tulsa, OK, USA). To ascertain the significant differences between samples, a Tukey mean comparison test set as the post hoc test was performed, with a *p*-value of <0.05 to indicate statistical significance.

## 3. Results and Discussion

### 3.1. Visual Appearance of the Films

**Figure 3:**
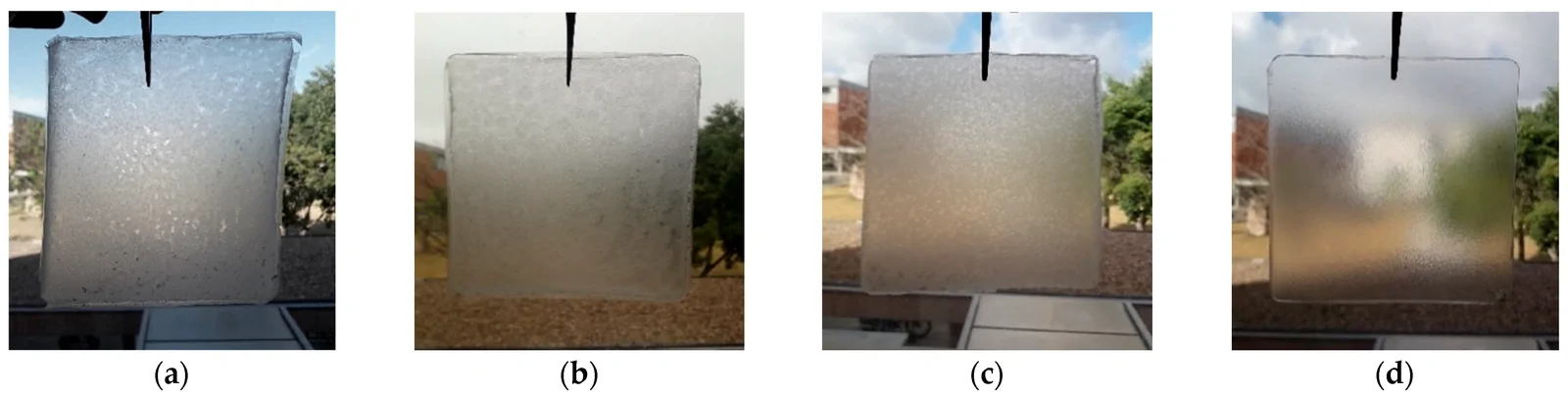
Photographs of all the films obtained. (**a**) Cnt film. (**b**) P film. (**c**) E film. (**d**) P+E film.

After the removal from the plexiglass plates, the resultant films were photographed to compare visual differences between them and conclude if the type of treatment that the cellulose pulp was submitted influenced the resultant film. Photographs of the obtained films are presented in Figure 3.

In general, all the films exhibited a smooth and partially transparent appearance, devoid of any notable deformities or irregularities. However, there are evident differences in terms of visual characteristics among the various film types. The Cnt film displayed the lowest level of transparency, whereas the P+E film emerged as the most transparent. As for the P and the E films, no distinct visual dissimilarities were observed. Consequently, it can be inferred that if the objective is to have a more transparent film, the preferable choice would be the combined treatment of enzymes and HPP. Nevertheless, using only the HPP or enzyme treatment, performed solely, the obtained films already are slightly more transparent than the control film.

**Figure 4:**
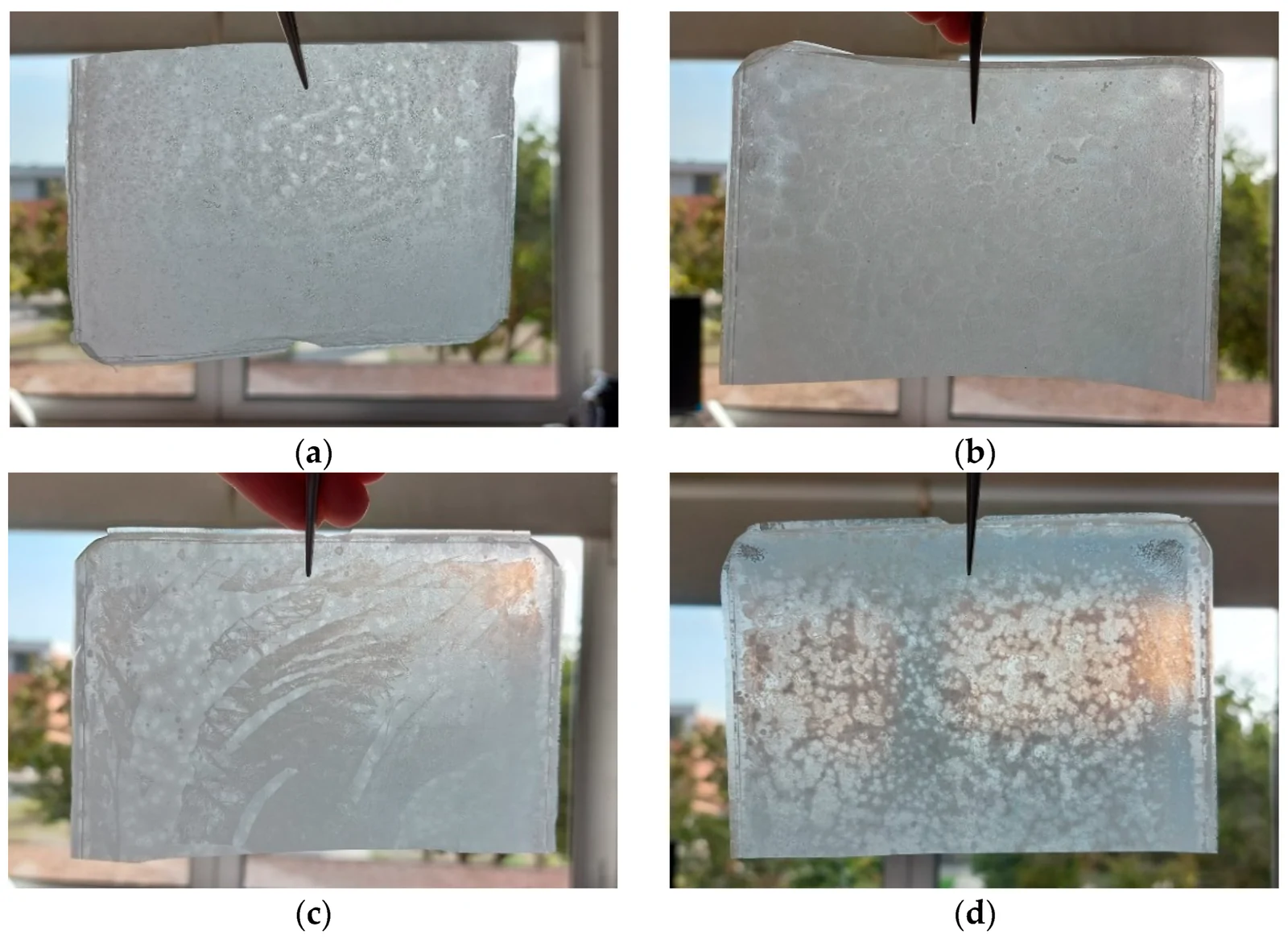
Photographs of all the films obtained four weeks after production. (**a**) Cnt film. (**b**) P film. (**c**) E film. (**d**) P+E film.

It should be emphasized that the photographs depicted in Figure 3 were captured immediately after the removal of the films from the plexiglass plates. However, upon analysis a few days later, it was observed that the films became whiter, cloudier, and less transparent, as illustrated in Figure 4.

This decline in visual transparency can be attributed to two factors. Firstly, the oxidation of cellulosic fibers may play a role, as certain groups such as -CO_2_, -CHO, and -COOH, which are present in these fibers (and increased during carboxymethylation [20], can bind with oxygen and generate compounds that affect the apparency of the films [21]. Secondly, research conducted by Kramar et al. (2023) [22] and Mei et al. (2021) [23] indicates that an increase in humidity conditions can lead to a decrease in visual transparency and as will be explained further, both treatments applied to the cellulose pulp result in an increase in the moisture contents of the cellulose films.

### 3.2. Film Analysis

#### 3.2.1. Moisture and Solubility

**Figure 5:**
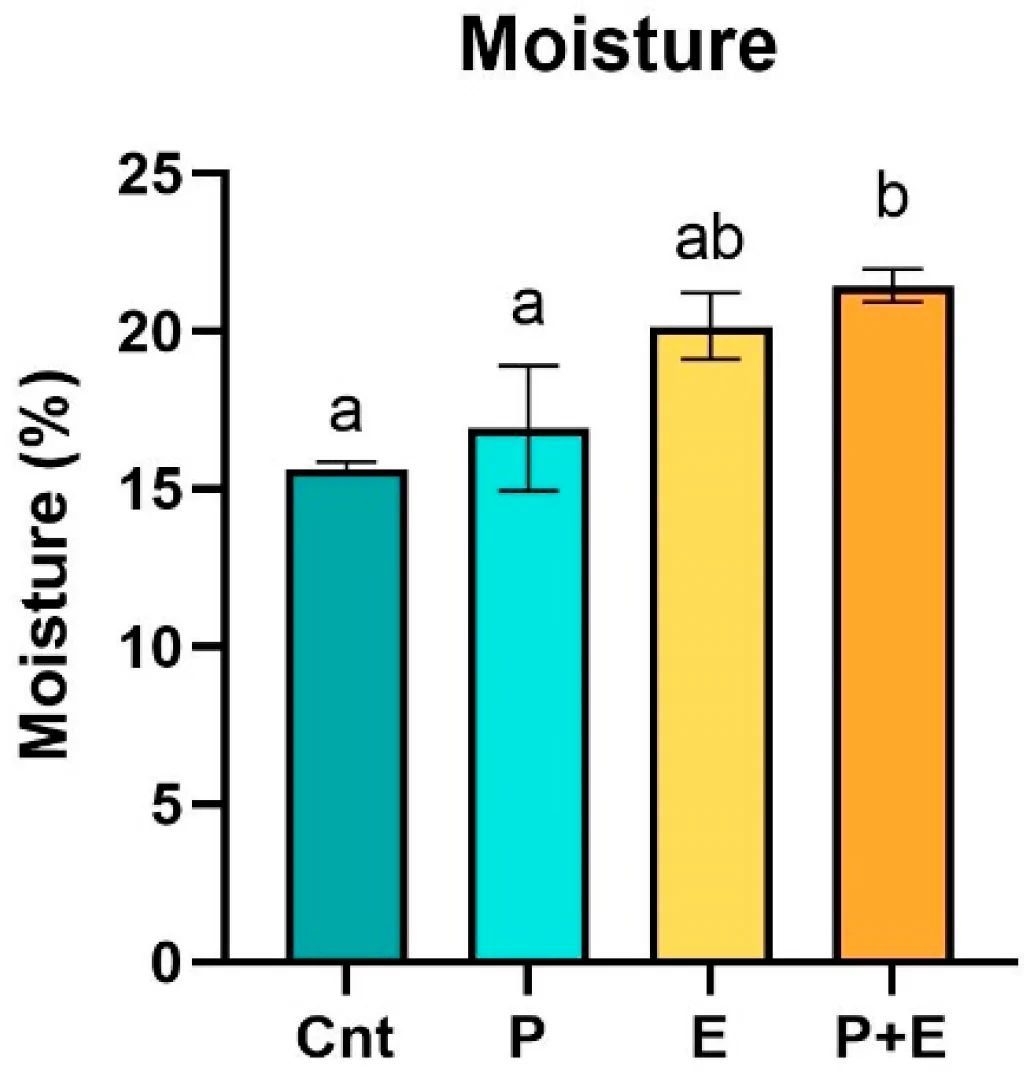
Moisture content (%) of the films stored at 53% RH for 3 days, at room temperature. Each column of the graph was expressed as the mean with error bars that represent the standard deviation (n = 3). The different lowercase letters on top of the columns (a, ab, b) indicate significant statistical differences (*p* < 0.05).

After 3 days of stabilization under controlled moisture conditions (53% RH) at room temperature, the films were cut into squares, and their moisture content was measured and calculated through Equation (1). The obtained values are presented in Figure 5.

The Cnt film showed the lowest moisture content (15.66 ± 0.20%) and the P+E film displayed the highest moisture content (21.45 ± 0.52%). This rise in moisture levels in the P+E film could potentially be attributed to the impact of HPP. Previous research conducted by Figueiredo et al. (2010) [13] has indicated that HPP enhances the accessibility of the cellulosic fibers, allowing a greater infiltration of water molecules into cellulose. Consequently, this increased water penetration on the structure of the film, may result in higher moisture percentages compared to films where no HPP treatment was performed. Additionally, Castañón-Rodríguez et al. (2013) [12] have also reported that HPP could lead to the formation of cracks in the cellulose structure, which in turn may contribute to higher rates of water absorption by cellulose and consequently by cellulose films. This increase in the moisture content might be a disadvantage if the films were to be applied to the food packaging area because it is desired that the packaging materials have a low moisture content to protect food from the surrounding atmosphere [24]. On the other hand, a higher moisture content might be advantageous in other areas, such as the use of cellulose materials in wound healing, where a higher constant moisture content is desired [25].

#### 3.2.2. Wettability and Water Vapor Permeability

**Figure 6:**
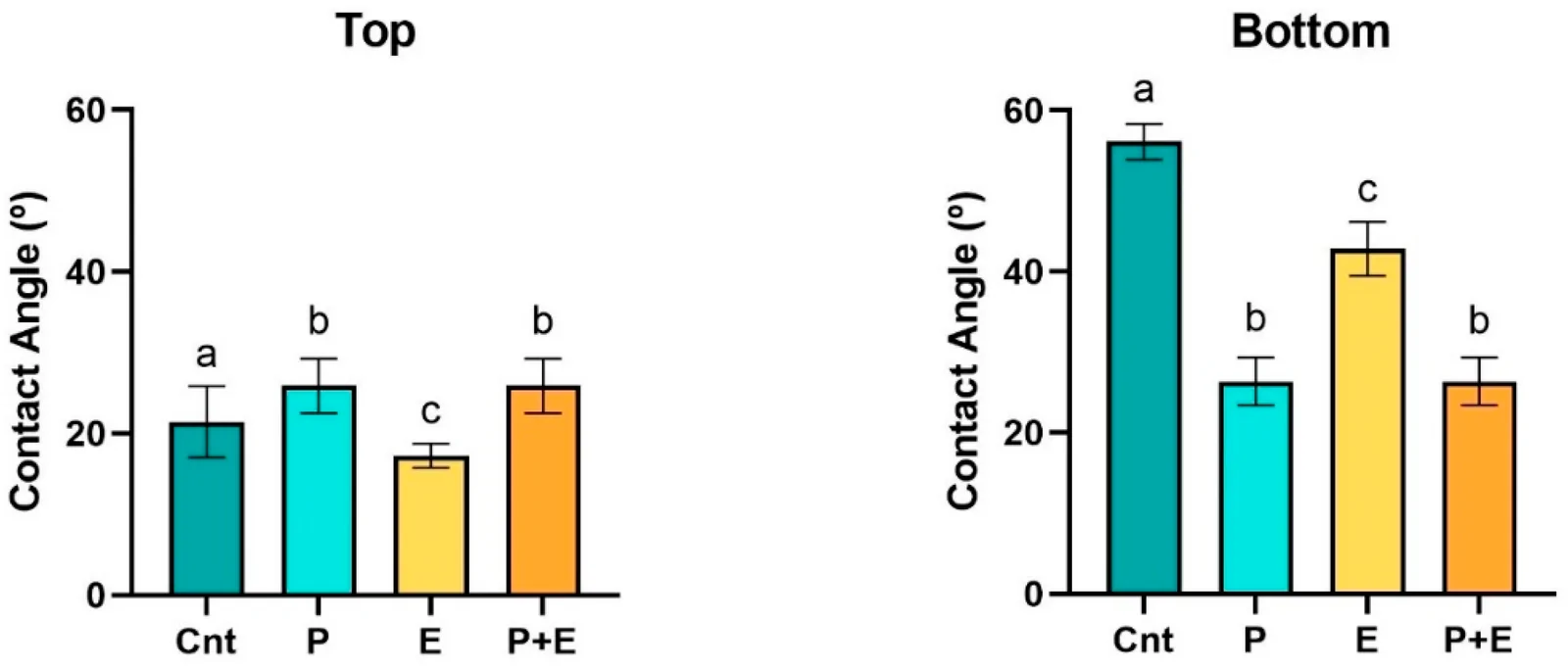
Contact angle of cellulose films. Each column of the graph was expressed as the mean value with error bars that represent the standard deviation (n = 18). The different lowercase letters on top of the columns (a, b, c) indicate significant statistical differences (*p* < 0.05).

The assessment of wettability for solid surfaces such as films and membranes relies on the crucial parameter known as the contact angle (CA) [26]. To determine the wettability characteristics, the contact angles were measured on both the top surface (which was directly exposed to the drying heat) and the bottom surface (which remained unexposed directly to heat). The results obtained from these measurements are depicted in Figure 6.

According to the literature, the CA of a surface can exceed 90°, if the surface is hydrophobic, or be less than 90°, if the surface is hydrophilic [27]. This implies that both sides of all cellulose films studied in this work are hydrophilic. However, variations exist between the different sides of the films and within the same side, depending on the type of film. Notably, the CA of the bottom side of the Cnt and E films is higher than the top side, indicating that the bottom side of these films is more hydrophobic than the top one. Furthermore, the CA values differ across the different sides of the films, depending on the type of treatment undergone by the cellulose pulp. On the top side of the films, it is evident that the P and P+E films exhibit slightly higher CA values, and the E film demonstrates a slightly lower CA value compared to the Cnt film. Conversely, on the bottom side, the CA values of the P, E, and P+E films are all lower than the Cnt film.

The differences in CAs between the top and bottom surfaces may be partly related to differences in their surface morphology, as the bottom surface was in direct contact with the plexiglass casting plate, whereas the top surface was exposed to the drying environment, which could have resulted in different degrees of roughness and surface organization [22]. However, surface roughness was not quantitatively measured by profilometry or AFM in the present study. Therefore, the contribution of roughness to the observed wettability differences remains hypothetical and cannot be confirmed from the contact-angle measurements alone. The lower CA values of the P and P+E films at the bottom side do not align with the findings reported in the literature. For instance, in the study conducted by Gonçalves et al. (2020) [28], although the studied films were not made from carboxymethylated cellulose, it was found that cellulose films treated by HPP tend to have higher Cas compared to control films. It was suggested that this discrepancy might be due to alterations in the intermolecular forces within the film caused by HPP. These alterations could result in a higher equilibrium of these forces compared to the forces between water and the films, thereby reducing the interaction between the water droplet and the film and, subsequently, reducing the CA. Furthermore, it was observed that the E films exhibited lower CA values than the Cnt films on both sides. This could be explained by the fact that enzymes break down the cellulose chains and expose different sites of cellulose [29]. This increased exposure of cellulose sites could enhance interactions with water, reducing the CA of the resulting cellulose films.

**Figure 7:**
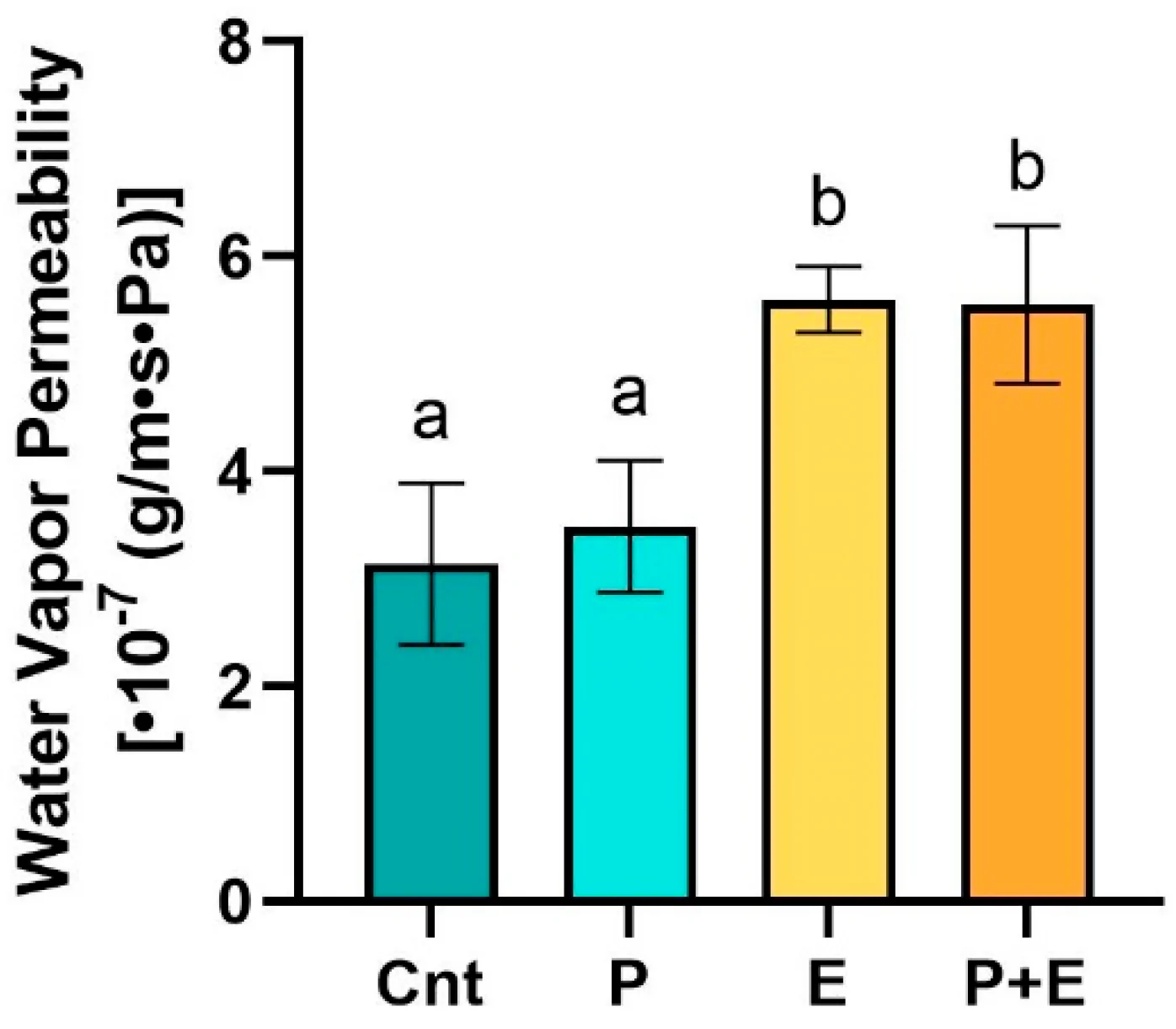
WVP of cellulose films. Each column of the graph was expressed as the mean value with error bars that represent the standard deviation (n = 3). The different lowercase letters on top of the columns (a, b) indicate significant statistical differences (*p* < 0.05).

Another important property in biodegradable films is the water vapor permeability (WVP). The WVP of a film or membrane indicates the amount of water vapor that passes through a film per unit of time, area, and pressure difference [30]. As such, the WVP of the produced films was measured and the results obtained are presented in Figure 7.

As can be seen in Figure 7, all different cellulose films have different WVP values. Notably, the Cnt film has the lowest WVP value (3.135 × 10^−7^ g/m·s·Pa), while the P+E film exhibits the highest WVP value (5.550 × 10^−7^ g/m·s·Pa). However, upon conducting an ANOVA analysis, it can be concluded that there is no significant statistical difference between the Cnt/P films and E/P+E films. Only the WVP values of the Cnt/P films and E/P+E films display statistical dissimilarity, suggesting that the enzymatic treatment significantly increases WVP, while HPP alone does not affect it.

The work conducted by Fu et al. (2018) [31] also confirmed the increase in WVP when an enzymatic treatment with cellulase was performed. In their research, the objective was to produce m-aramid/cellulose membranes using an enzymatic degradation method, where the membranes were enzymatically treated with cellulase after production. The results revealed that the water transmission across the membrane increases with enzymatic degradation. The WVP results also align with the moisture results (Figure 5), where the films with higher moisture contents (E and P+E) also exhibited higher WVP. The permeation of water through materials such as membranes or films involves adsorption followed by the diffusion of water from one side to the other [31]. Therefore, if the E and P+E films tend to adsorb higher moisture contents when stored for the same period and at the same conditions as the other films (Cnt and P), this could be reflected in higher WVP, as observed in Figure 7. The CA measurements also support the WVP values of the films. The enzymatic treatment was found to be associated with a reduction in CA (increase in hydrophilicity), which indicates a higher affinity for water. This increased contact with water may contribute to an increase in water permeation, ultimately resulting in higher WVP.

From a food-packaging perspective, the increased moisture content and WVP observed particularly in the E and P+E films may represent a limitation for their use as stand-alone packaging materials. The P+E film exhibited an approximately 1.8-fold higher WVP and a 1.4-fold higher moisture content than the Cnt film. Higher WVP facilitates the transfer of water vapor through the packaging material and may therefore be unsuitable for moisture-sensitive foods, for which limiting moisture gain or loss is essential to preserve texture, stability, and overall quality. Nevertheless, the suitability of a packaging material depends on the requirements of the intended food product. These films could potentially be considered for products requiring less restrictive moisture barriers or as a structural layer in multilayer packaging systems. Further optimization, including the incorporation of hydrophobic compounds, surface coatings, or combination with materials presenting better moisture-barrier properties, would be necessary before their application in moisture-sensitive food packaging.

#### 3.2.3. Mechanical Properties

**Figure 8:**
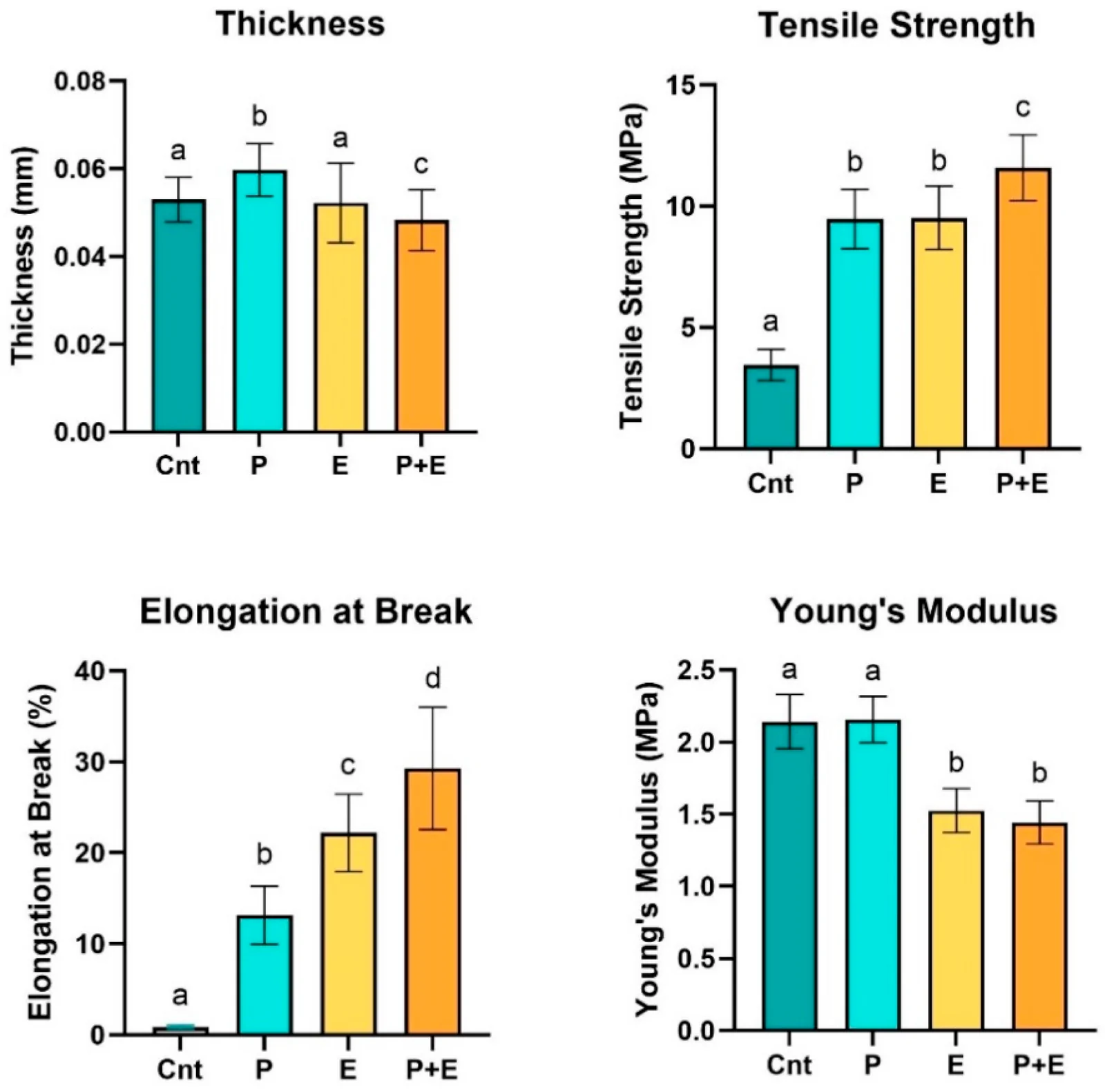
Mechanical properties of cellulose films (Thickness, TS, EAB, YM). Each column of the graph was expressed as the mean value with error bars that represent the standard deviation (n = 10). The different lowercase letters on top of the columns (a, b, c, d) indicate significant statistical differences (*p* < 0.05).

Parameters such as tensile strength (TS), elongation at break (EAB), and Young’s modulus (YM) are frequently used to evaluate the mechanical properties of films, which are dependent on the thickness of the films. As such, the thickness and the referred parameters of the films were measured, and the obtained results are presented in Figure 8.

Starting with the thickness of the cellulose films, it is possible to observe that there are slight variations between them, being the P film exhibited the highest thickness (0.060 ± 0.004 mm) and the P+E film the one with lowest (0.052 ± 0.007), however no correlation was found between the variation in the films’ thickness and the treatments performed on the cellulose pulp. On the other hand, differences in TS values are observed depending on the type of film and the type of treatment that the cellulose pulp went through. The Cnt film exhibits a lower TS (3.420 ± 0.617 MPa) while the P+E film demonstrates a higher value (9.519 ± 1.306 MPa), resulting in a 2.7-fold increase in the TS of the cellulose films produced with HPP and cellulase. Notably, the EAB values show significant differences among the various treatments, in a similar trend to TS. Although all treatments appear to enhance the elongation of the films, the P+E film exhibits the highest value (29.294 ± 6.725%) while the Cnt film displays the lowest value (0.824 ± 0.209%). This indicates that the combined HPP and enzymatic treatment leads to a 35.5-fold increase in the elongation of the cellulose films. For a better understanding of these results, Video S1 presents in the Supplementary Materials shows examples of the mechanical tests performed in all films. Additionally, variations in the YM of the cellulose films are observed. The Cnt and P films exhibit higher YM values compared to the E and P+E films, suggesting that the enzymatic treatment may lead to a reduction in the YM value.

Figueiredo et al. (2010) [13] conducted a study that observed an increase in the TS of eucalyptus pulp after subjecting it to HPP at 400 MPa for 10 min at room temperature). They proposed that this increase could be attributed to a rearrangement of the cellulosic fibrils matrix, resulting from the aggregation of crystallites. This rearrangement resulted in stronger fibril force, which could potentially explain the verified increase at the final TS of the cellulose films. However, the increase in TS cannot be solely attributed to the HPP treatment. In the E film, the TS was also higher than in the Cnt film, which aligns with the findings of Efrati et al. (2013) [32]. In their study, enzymatic treatment with cellulase was found to increase the TS of cellulose pulp. The authors suggested that the enzymatic treatment with cellulase of the cellulose pulp might lead to a more compact fibril matrix with closer contact between the cellulosic fibrils, which could potentially explain the increase in the TS observed in materials produced from this pulp, such as the E film. The increase in the EAB values is also linked to both the HPP and enzymatic treatments, individually or combined. Gonçalves et al. (2020) [28] also observed an increase in the EAB of acetate cellulose films treated with HPP. According to Figueiredo et al. (2010) [13], this increase could be attributed to enhanced water penetration in previously inaccessible surfaces, resulting in a higher amount of strongly bonded water in the cellulose matrix and consequently, an increase in the elongation of the pulp. The increase in the EAB in the E film further supports the notion that the enzymatic treatment of the cellulose film contributes to an increase in the EAB of the final films. This finding aligns with the work of Efrati et al. (2013) [32], where an increase in the flexibility of the cellulosic fibers was observed after cellulase treatment of the cellulose pulp, which might explain the increased EAB of the E film. Additionally, the rise in the EAB of the films could also be attributed to the escalation in moisture content observed earlier, particularly for the E and P+E films. It is well-known that water acts as a plasticizing agent [33], which can enhance the flexibility of the film and, consequently, its EAB. Furthermore, the decline in the YM of the E and P+E films can be primarily attributed to the enzymatic treatment. Although this reduction has not been documented in any available studies, as mentioned earlier, cellulases are responsible for breaking down the cellulosic fibers [29]. This degradation could potentially diminish the stiffness and structural integrity of the cellulose matrix, resulting in a decrease in the YM of the E and P+E films. On the other hand, this decrease may also be linked to the increase in moisture content in both E and P+E films, as observed in the study conducted by Sahputra et al. (2019) [34]. According to their findings, an increase in moisture content can transform polymers from brittle solids to smoother and more flexible polymers. This transformation occurs due to the additional water molecules near the polymer chains, which reduces the intermolecular forces between them and enhances molecular mobility.

The mechanical results should also be considered together with the moisture-content and wettability results. Although all films were conditioned and mechanically tested at approximately 53% RH, the E and P+E films retained more moisture than the Cnt and P films. Moreover, the E film exhibited lower contact angles on both surfaces, while the P+E film showed a lower contact angle than the Cnt film on its bottom surface, indicating a greater affinity for water. The retained water may act as a plasticizing agent by modifying intermolecular hydrogen bonding and increasing the mobility of the cellulose chains. This effect may have contributed to the higher EAB and lower YM observed for the enzymatically treated films.

Marcuello et al. [35] demonstrated at the nanoscale that increasing RH decreased the Young’s modulus of different lignocellulosic films. Nevertheless, crystalline cellulose nanocrystal films were comparatively less sensitive to moisture, exhibiting a 15.6% decrease in Young’s modulus between 15 and 95% RH, whereas substantially greater reductions were observed for the hemicellulose-based films. This indicates that the influence of moisture on mechanical behavior depends strongly on the composition, crystallinity, and structural organization of the material. In addition, Misaka et al. [36] demonstrated that water can be retained within the nanoscale spaces of dense three-dimensional cellulose-nanofiber networks and that this captured water contributes to the wettability of the films.

However, moisture cannot be considered the sole factor responsible for the mechanical behavior observed in the present study. In particular, the increase in TS of the E and P+E films indicates that structural modifications promoted by the enzymatic and HPP treatments, such as changes in fibril organization and interactions within the film matrix, also contributed to their mechanical performance. Since the films were not mechanically evaluated at different RH or moisture levels, the independent contribution of moisture cannot be quantitatively determined from the present results.

Adequate mechanical strength and extensibility are important requirements for the potential application of films in different areas. For example, packaging materials must withstand the mechanical stresses associated with manufacturing, handling, transport, and storage [37]. Therefore, the increases in TS and EAB observed after the HPP and enzymatic treatments indicate an improvement in the mechanical performance of the films.

Mechanical properties are also relevant for biomedical materials such as wound dressings. Zaman et al. [38] reported TS and EAB values of 12.7 MPa and 40.4%, respectively, for a wound-dressing material, which are of the same order of magnitude as those obtained for the P+E film. However, similarity in mechanical properties alone is insufficient to establish suitability for wound-healing applications. Parameters such as cytotoxicity, biocompatibility, swelling capacity, liquid and water-vapor management, antimicrobial activity, degradation behavior, and biological performance were not evaluated in the present study. Therefore, the results only indicate that the mechanical properties of the P+E film may justify further investigation to determine potential applications for these films.

#### 3.2.4. Color Parameters and Transparency

The optical properties of cellulose films were also evaluated. As such, the color parameters (L*, a*, b*) and transparency (calculated through the absorbance at 600 nm) were measured, and the results are presented in Table 2.

There are variations in the optical properties of cellulose films. The L* values indicate that all films are close to a whiter color, as their values are close to 100. The different types of treatment that the pulp underwent do not seem to have a significant influence on the L* values of the final films, as the values are similar. However, it is worth noting that the E film has a slightly lower L* value, suggesting that the enzymatic treatment may result in a reduction in the lightness of the films. In terms of the a* value, each film exhibits a different value. The P film has the highest value, while the P+E film has the lowest value. This indicates that the HPP treatment tends to shift the films towards a more neutral color, as the a* value approaches 0. However, the E and P+E films show an increase in greenness, as the a* value decreases, which suggests that the enzymatic treatment leads to greener films. Regarding the b* value, the enzymatic treatment has a negative impact on the yellowness of the E film. On the other hand, the HPP treatment, when performed solely, does not seem to affect the b* value. However, when combined with the enzymatic treatment, it mitigates the negative impact and moves the P+E film towards a less yellow color (lower b* value). Lastly, the transmittance values also exhibit differences. It is important to note that these measurements were taken three days after the production of the films, and as mentioned before, over time the films tend to become whiter and less transparent (as seen in Figure 4). This trend should be indeed investigated in future work to infer the reasons for this whitening effect. Nevertheless, analyzing the transmittance results allows the conclusion that the combined HPP and enzymatic treatment does not significantly impact the film transparency compared to the Cnt film.

Regrettably, there is a scarcity of research studies examining the impact of HPP or enzymatic hydrolysis on the optical properties of cellulose films, which makes it challenging to comprehensively analyze the verified effects. However, two noteworthy studies offer potential explanations for the observed outcomes. One such study, conducted by Gonçalves et al. (2020) [28], investigated the treatment of cellulose films with HPP. The findings indicate that HPP increased the L*, a decrease in the red (+a*) and yellow (+b*) colors, and an increase in the opacity of the films. These results align with some of the confirmed effects, such as the increase in the a* value and decrease in transparency caused by HPP when performed solely. On another hand, another study conducted by Vänskä et al. (2015) [40] may shed light on some of the effects observed following the cellulase treatment. According to this study, the cellulase treatment of cellulose pulps can introduce reducing end-groups in the pulp, which in the presence of the heat required (60 °C) during the enzymatic treatment, contribute to the yellowing of the cellulose pulp. This explanation may account for the increase in the b* value and the decrease in transparency of the films when the enzymatic hydrolysis is performed solely. However, no other studies were found that could elucidate the role of the enzymatic or HPP treatments on the optical properties of cellulose films about the remaining effects.

#### 3.2.5. Surface Morphology

**Figure 9:**
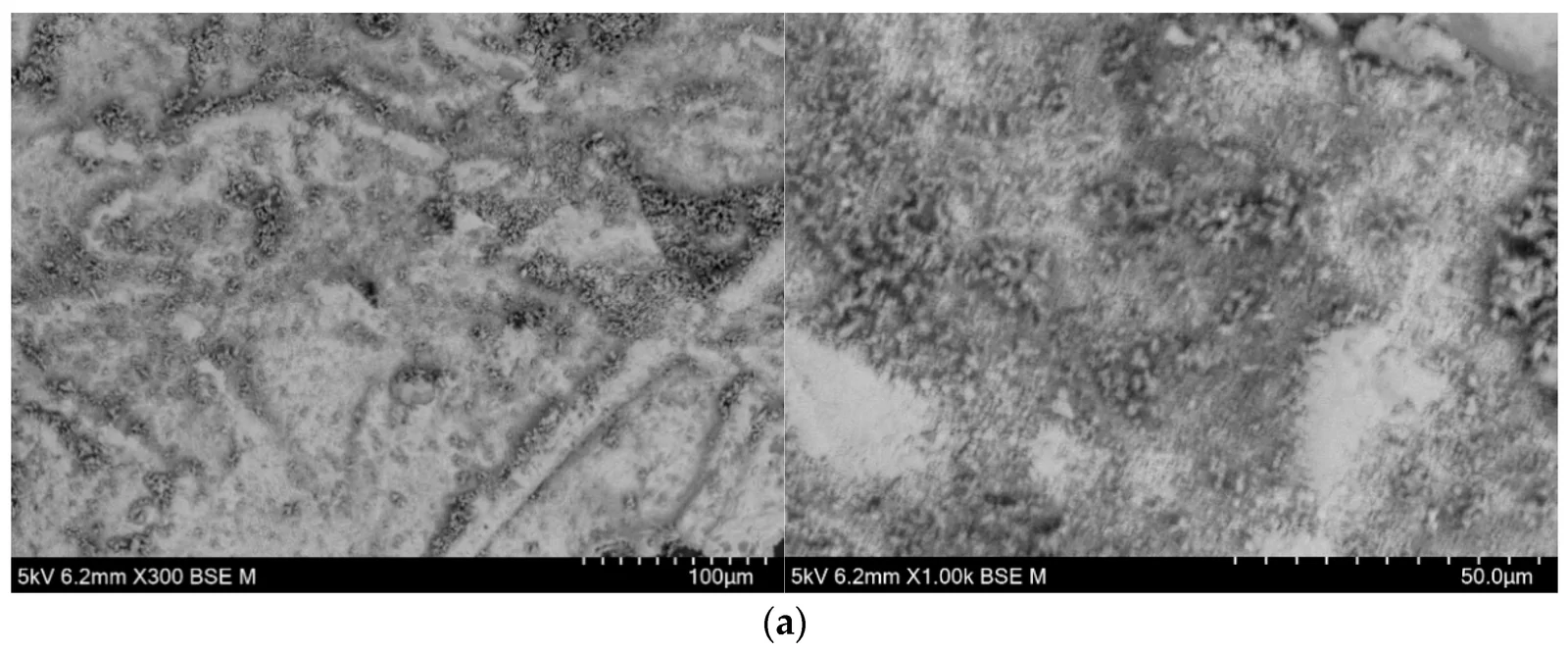

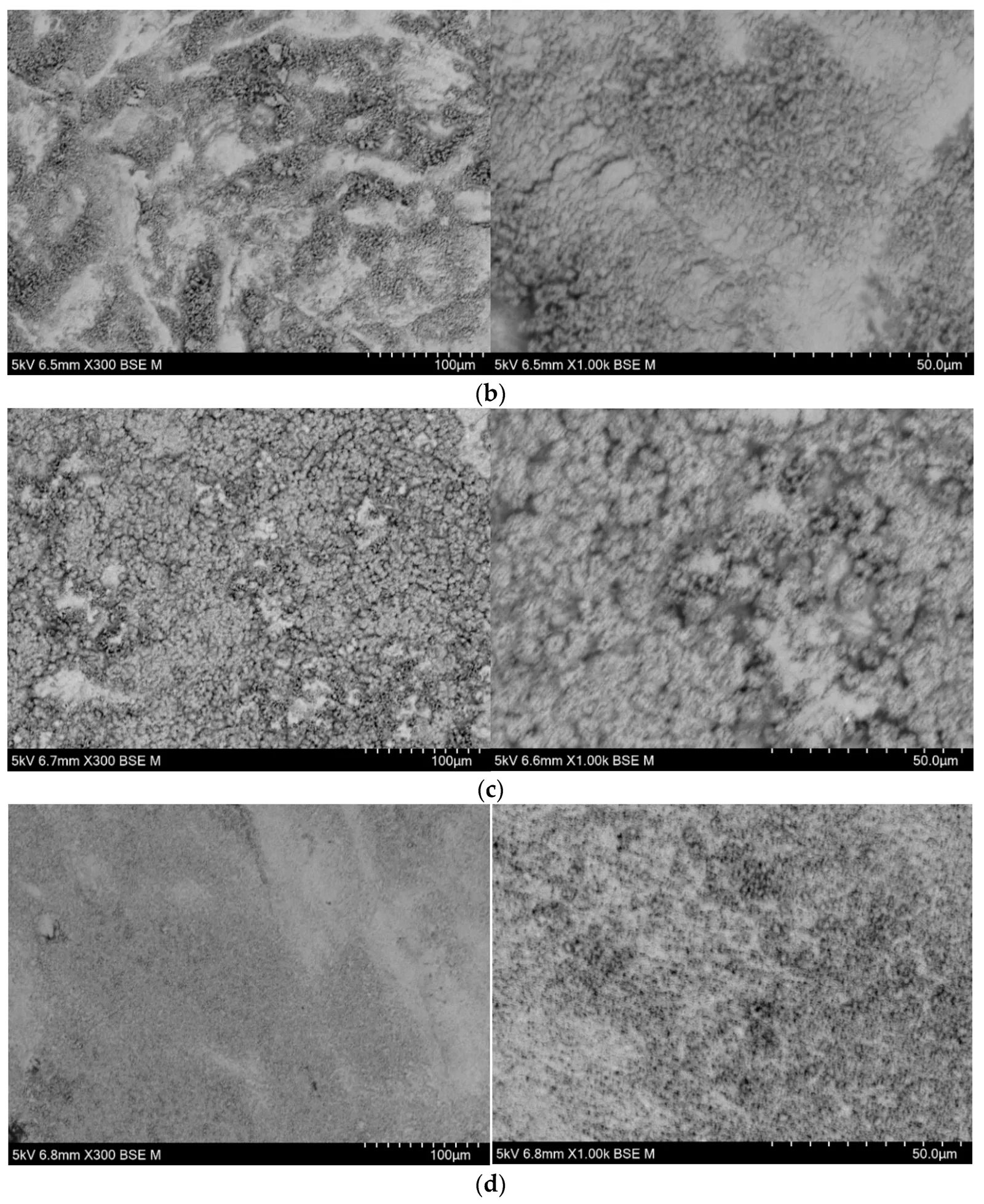
SEM images of the cellulose film surfaces. The pictures on the left represent the ×300 ampliation and the pictures on the right represent the ×1000 magnification. (**a**) Cnt film. (**b**) P film. (**c**) E film. (**d**) P+E film.

To have a closer evaluation of the morphology of the film surface, SEM images of the cellulose films were recorded and are present in Figure 9.

Qualitative differences were observed among the surfaces of the cellulose films. The P+E film exhibited a comparatively smoother and more uniform surface, whereas the Cnt, P, and E films showed rougher and more heterogeneous regions. The P film appeared slightly rougher than the Cnt film, although this difference was relatively subtle, while the E film exhibited a more pronounced porous and granular appearance. These observations suggest that the pretreatments may have influenced the organization of the cellulose matrix during film formation.

The slightly rougher appearance of the P film may be related to pressure-induced structural modifications in cellulose, since HPP has been reported to alter the organization of cellulose fibers and promote the formation of pores or void spaces [11]. Nevertheless, the SEM images alone do not provide direct evidence that these mechanisms occurred in the present films.

Similarly, the smoother surface observed for the P+E film may hypothetically be associated with changes in cellulose accessibility and enzymatic action induced by HPP. Previous research has shown that high pressure can enhance enzymatic reactions in cellulosic fibers [15]. It is therefore possible that HPP promoted a more extensive or more uniform enzymatic modification of the pulp, contributing to improved organization of the material during casting and drying. Conversely, a less uniform enzymatic action could potentially have contributed to the heterogeneous surface observed for the E film.

However, particle size, fibrillation, fiber morphology, and the extent and uniformity of enzymatic hydrolysis were not directly evaluated in the present study. Consequently, these explanations remain hypotheses and cannot be confirmed from the SEM images alone. Additional characterization of the treated cellulose pulp would be required to establish the mechanisms responsible for the observed surface morphologies.

#### 3.2.6. Thermogravimetric Analysis

**Figure 10:**
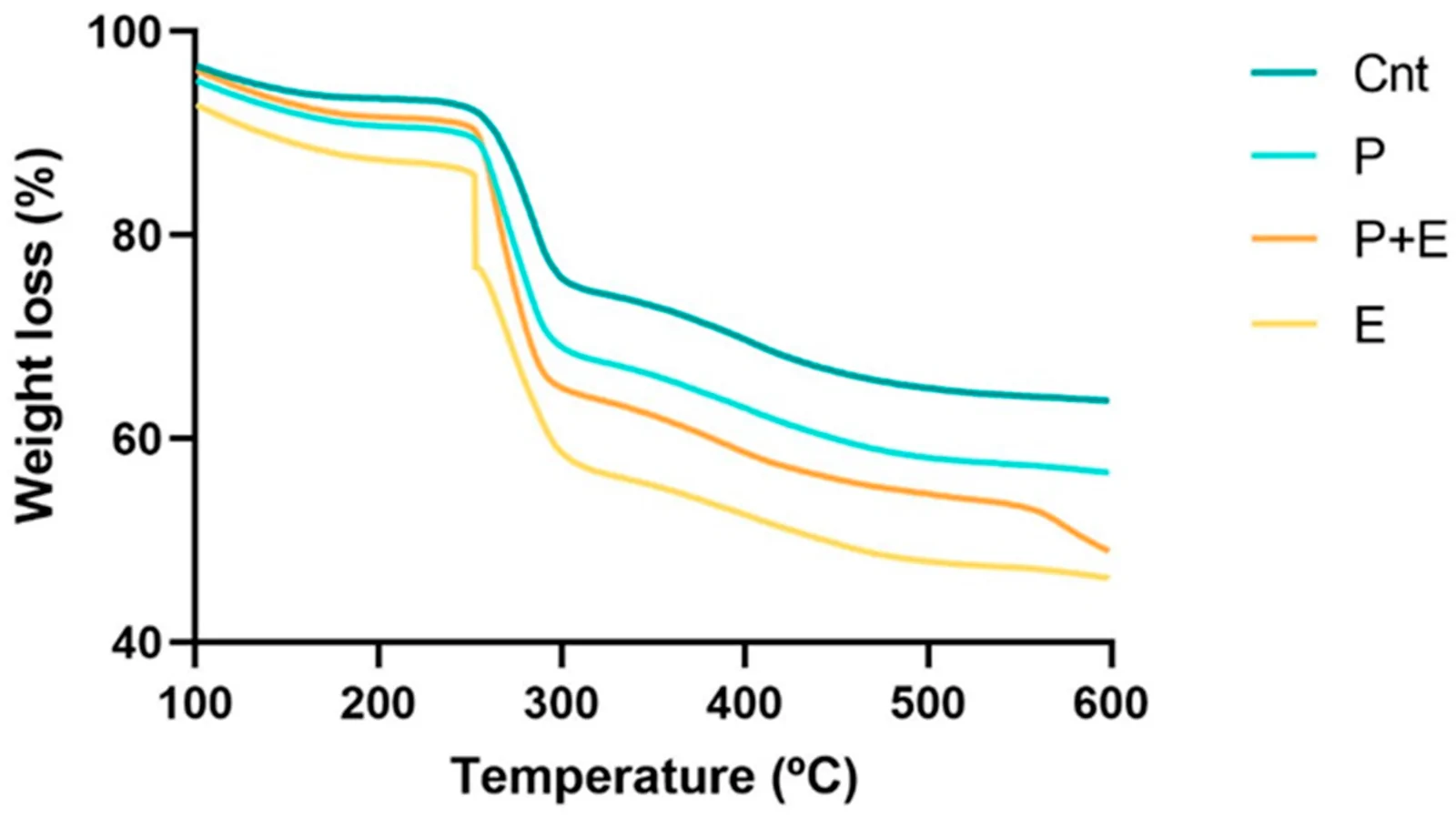
Thermogravimetric analysis (TGA) curves of all cellulose films.

TGA is a methodology that allows the evaluation of the thermal stability of materials. This practical approach is based on the decomposition of materials by heating, leading to the breakage of the bonds that maintain the structure of the molecules [20]. As such, the analysis was performed in all different cellulose films and the resultant TGA curves are present in Figure 10.

All TGA curves exhibit similar patterns, displaying three stages of degradation for all films, except for the P+E film, which exhibits an additional fourth degradation stage. The initial stage of degradation for all films, occurring at temperatures ranging from 100 to 200 °C, is attributed to the loss of volatile compounds and water molecules (dehydration process) [41]. The second stage, observed between 200 and 300 °C corresponds to most of the degradation of carboxymethylated cellulose (CMC), reported in the literature to occur within the temperature range of 200–340 °C [20]. The third stage (300–450 °C), also observed in all films, may be associated with the degradation of CMC molecules not depredated in the previous stage. Finally, the fourth and final stage (550–600 °C), exclusively observed in the P+E film could be linked to the complete carbonization of the sample [20]. The fact that this stage is only observed in the P+E film suggests that the combined HPP and enzymatic treatment reduces the thermal stability of the cellulose films at very high temperatures (>500 °C).

In general, all various treatments appear to decrease the thermal stability of the films. Within the same range of temperatures, the P, E, and P+E exhibit higher rates of weight loss compared to the Cnt film. Previous research has demonstrated that the hydrogen bonds found in the cellulose structure contribute significantly to its unique properties, including its thermal stability, where the hydrogen bonds act as a buffer that maintains the cellulose stability in a wide range of temperatures [42]. Since HPP mainly affects the hydrogen bonds of the processed materials [12], this might explain the reduced thermal stability of the P film compared to the Cnt film, as HPP may break or modify the hydrogen bonds in cellulose, leading to a decrease in thermal stability and, consequently, reducing the thermal stability of the produced cellulose films. On the other hand, the cellulase hydrolysis treatment targets the glycosidic bonds that bind the glucose units and form the cellulose chains [29] and by breaking these bonds, one of the noticeable effects is a reduction in the molecular weight of cellulose. A study conducted by Calahorra et al. (1989) [43] demonstrated that the thermal stability of cellulose increases with an increase in its molecular weight. Therefore, it can be deduced that cellulose with lower molecular weight has lower thermal stability. Since cellulase treatment reduces the molecular weight of cellulose, it is expected that the thermal stability of cellulose would also be reduced, which might explain the reduced thermal stability of the E film. Considering these factors, the P+E film, obtained through the combined application of HPP and enzymatic treatment, would be expected to exhibit the lowest thermal stability. However, as shown in Figure 10, its degradation curve lies above that of the E film, suggesting that the E film may have lower thermal stability. Nevertheless, the P+E film also displays a fourth degradation stage, which may indicate that it has the lowest thermal stability among the films. In terms of practical applications, for example for the implementation of the cellulose films in the food packaging area, materials used as packages must have good thermal properties. The materials used in food packages must be resistant to heat to allow the transport and storage of the foods [44]. The produced cellulose films do not present any significant degradation until around 200 °C, a range of temperature to which foods are not usually submitted, so it might be possible to affirm that in terms of the thermal properties these cellulose films might be good candidates to be used as food packaging materials; however, it was not tested the resistance of these films to refrigeration temperatures and so it is not possible to affirm that they could applied as food packaging materials for refrigerated foods.

#### 3.2.7. X-Ray Diffraction

**Figure 11:**
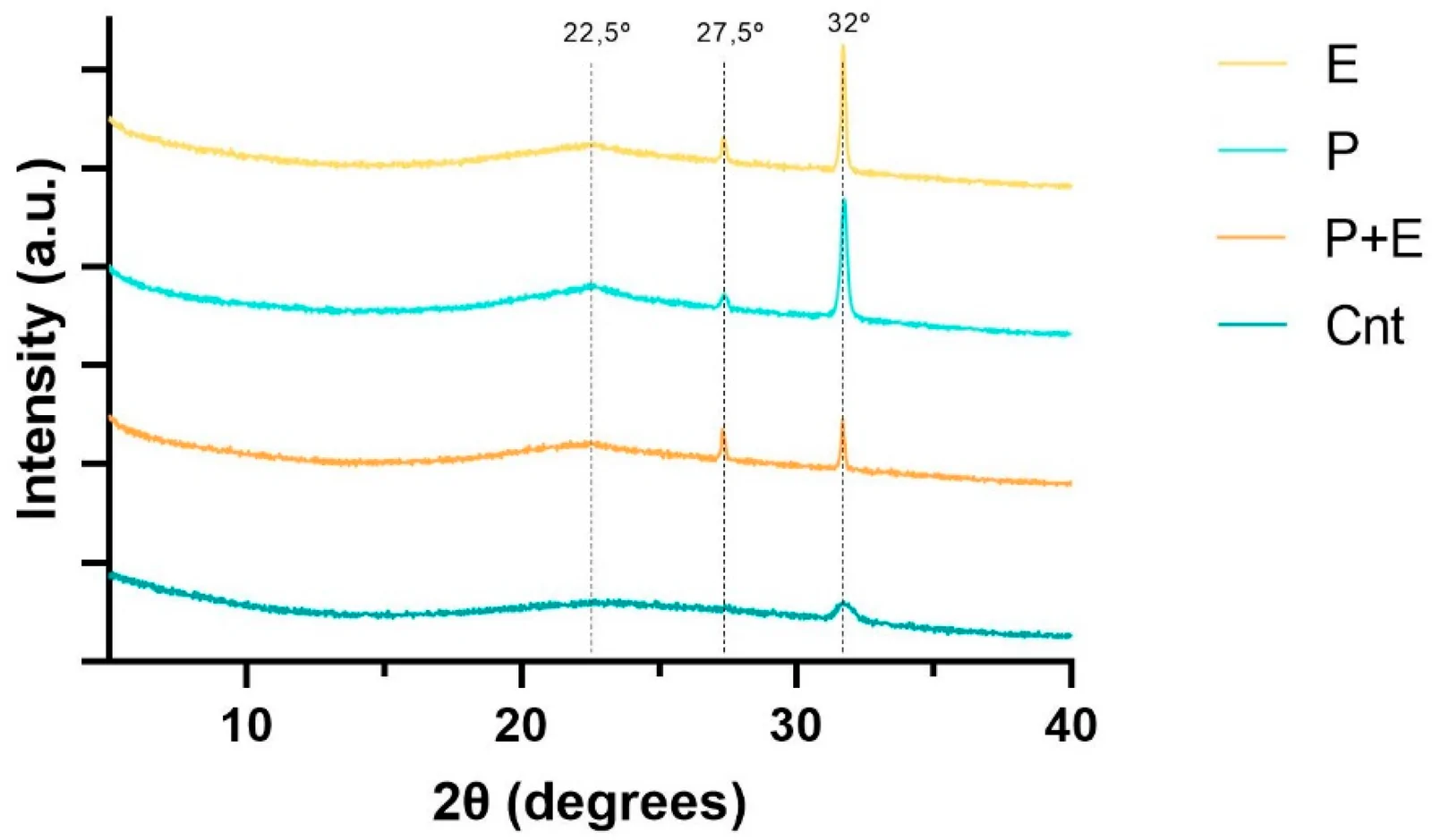
X-ray diffractograms of all cellulose films.

To evaluate differences in the crystallinity of the cellulose films, the XRD patterns of the films were combined in one graph, presented in Figure 11.

According to Figure 11, the XRD patterns of all films remained unchanged, displaying three distinct crystalline peaks. These peaks were observed at approximately 22.5°, 27.5° (both barely visible in the Cnt film), and 32°, which could be attributed to the crystal structures of cellulose I and cellulose II. Although the XDR patterns were consistent across the films and the three peaks appeared to be located at the same positions, their intensities varied depending on the treatment applied to the pulp. Upon analyzing the XDR patterns, the crystallinity index of the films was estimated to be 9.07% for the P+E film, 3.64% for the E film, 3.15% for the P film, and 1.37% for the Cnt film. These results indicate that all treatments led to an increase in the crystallinity of the cellulose films, compared to the Cnt film.

Although the increase in CrI from 1.37% for the Cnt film to 9.07% for the P+E film corresponds to a 6.6-fold relative increase, the absolute increase was 7.70 percentage points, and the CrI values of all films remained relatively low. Therefore, the result should be interpreted as a relative increase in the proportion or ordering of crystalline domains within a predominantly amorphous film matrix, rather than as extensive crystallization of the material. Moreover, XRD-derived crystallinity indices are influenced by the calculation method, peak deconvolution, crystallite orientation, and sample preparation. Consequently, the values obtained in the present study are most appropriately used for comparative purposes among films analyzed under the same conditions rather than as absolute measurements of crystalline content [45].

Several non-mutually exclusive mechanisms may account for the higher CrI values of the treated films. Figueiredo et al. [13] reported that the treatment of eucalyptus pulp at 400 MPa promoted the growth of crystalline domains predominantly through lateral aggregation or co-crystallization of neighboring cellulose crystallites and, to a lesser extent, through the incorporation of cellulose chains from non-crystalline domains. The authors also reported increased swelling and accessibility of amorphous cellulose regions following HPP. Such pressure-induced rearrangements may have been partly retained during the subsequent processing steps and may explain the higher CrI of the P film compared with the Cnt film.

The increase observed for the E film may instead be mainly associated with the preferential action of endocellulase on accessible and less ordered cellulose regions. Enzymatic hydrolysis of part of the amorphous fraction can result in a relative enrichment of the residual crystalline domains, increasing the calculated CrI even when no substantial formation of new crystalline material occurs [46,47]. This distinction is important because an increase in relative crystallinity does not necessarily indicate that the total amount of crystalline cellulose increased; it may also result from the preferential removal or disruption of non-crystalline material.

The highest CrI observed for the P+E film may therefore result from the combined action of these mechanisms. HPP may have promoted fibril rearrangement and aggregation while simultaneously increasing the accessibility of amorphous domains to the enzyme. The subsequent endocellulase treatment may then have preferentially hydrolysed part of these accessible, less ordered regions, leading to a greater relative contribution of the remaining ordered domains. This combined effect could explain why the increase observed for P+E was greater than that observed when either treatment was applied individually.

Nevertheless, the possible influence of the subsequent carboxymethylation reaction must also be considered. The introduction of carboxymethyl groups generally disrupts the native hydrogen-bonding network and crystalline organization of cellulose [48]. At the same time, changes in cellulose accessibility caused by HPP or enzymatic hydrolysis may influence the extent and distribution of carboxymethyl substitution.

Therefore, differences in the degree of substitution among the samples could have contributed to the XRD patterns of the resulting materials. Since the degree of substitution, molecular weight, and carboxyl content of the produced CMC were not determined, the contribution of the carboxymethylation step cannot be distinguished from the structural effects directly induced by HPP and enzymatic treatment.

Film formation may also have influenced the final degree of molecular ordering. During casting and drying, solvent evaporation allows polymer chains to approach each other and reform intermolecular hydrogen bonds. Although the same water/ethanol composition and drying conditions were used for all samples, possible pretreatment-induced differences in chain length, substitution pattern, dispersion, hydration, and molecular mobility could have affected chain packing during film formation. Therefore, the higher CrI of the P+E film most likely reflects the combined influence of pressure-induced structural rearrangement, preferential enzymatic modification of accessible amorphous domains, possible differences arising during carboxymethylation, and chain reorganization during casting and drying. The present XRD results do not allow the individual contribution of these mechanisms to be quantified, and further characterization of the intermediate CMC would be required to establish a definitive mechanistic explanation.

#### 3.2.8. FTIR Analysis

**Figure 12:**
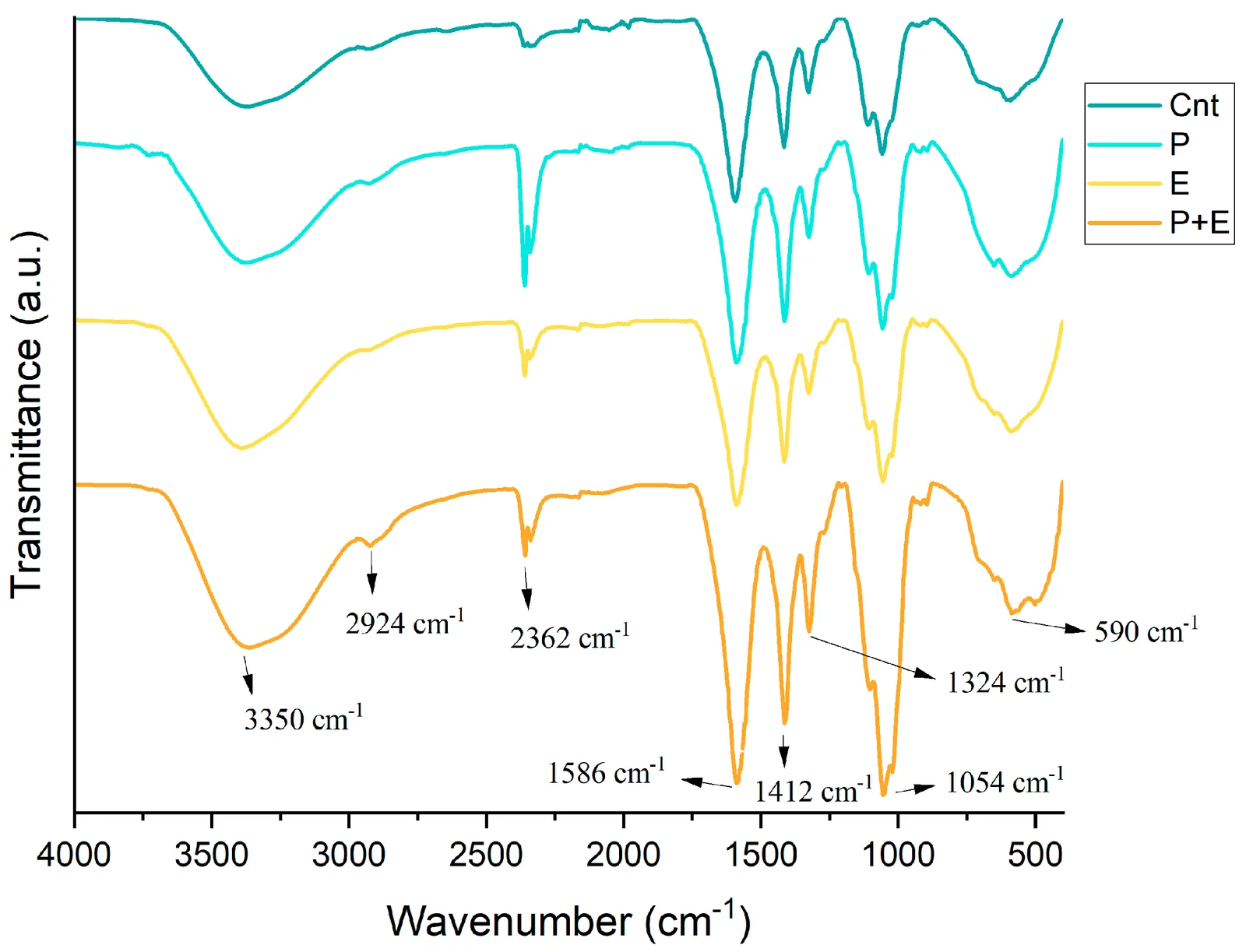
FTIR spectrum of all cellulose films.

To observe potential different interactions and different intensities of interactions between the different cellulose films, the FTIR spectra for all four films were obtained and are presented in Figure 12.

Cellulose films exhibit identical characteristic absorption bands at specific wavelengths, however, their intensities vary depending on the film. Table 3 provides a comprehensive overview of the eight distinct absorption bands associated with each cellulose film and their corresponding chemical groups.

In general, the intensity of an absorption band observed on an FTIR curve is directly related to the number of bonds that absorb at a specific wavelength. Therefore, an absorption band that corresponds to a higher number of bonds will exhibit a higher intensity. By understanding this principle, it becomes possible to draw some conclusions about the variations in intensities of absorption bands among the different films. Starting with the first band (3350 cm^−1^), it is evident that the E film and especially the P+E film have higher intensities. According to Lombo Vidal et al. (2020) [49] this band is sensitive to hydrogen bonds, and changes in its intensity are usually related to the water content and its interaction within the film matrix [49]. Therefore, it is logical that the E and P+E films exhibit higher intensities for this band, as they also showed higher moisture content. In the second band (2924 cm^−1^), a slightly higher intensity is verified for the P+E film, what might be related to the carboxymethylation of cellulose. This process results in the addition of -CH_2_COOH groups to the cellulose structure [20], increasing the number of C-H groups, which, consequently, might be reflected as an increase in the intensity of the FTIR band. As mentioned in Table 3, the third band (2362 cm^−1^) corresponds to the atmospheric carbon dioxide. Thus, the observed differences in intensity for this band are not related to the film composition but rather to the environmental conditions during the analysis. Consequently, the higher intensity observed in the P film can be attributed to a higher atmospheric carbon dioxide content during the analysis. Moving on to the fourth band (1586 cm^−1^), it is evident that the P+E film has a significantly higher intensity compared to the other films. According to Ramli et al. (2015) [51], this band indicates the presence of the carboxyl groups (COO^−^), which suggests that the hydroxyl groups were substituted by carboxyl groups during the carboxymethylation process. Therefore, the higher intensity observed in the P+E film suggests that HPP facilitated a more intense carboxymethylation process, resulting in a higher number of substituted hydroxyl groups and a higher concentration of carboxyl groups. Figure 12 also suggests that the bands at 1412, 1324, and 1054 cm^−1^ are not affected by HPP, which leaves only the possibility of the enzymatic treatment or the carboxymethylation process as possible factors influencing the bonds/groups that these bands correspond to. The enzymatic treatment with cellulase breaks down the glycosidic bonds linking the glucose units and forming the cellulose chains [29]. Although this treatment should not directly create C-H bonds or groups, it may expose some of these bonds/groups in the resultant glucose units leading to an increase in band intensity. HPP promotes higher enzyme accessibility [15], which could explain why only the P+E film show a significant increase in the intensity of these three bands. As mentioned before, the carboxymethylation process, consists of the addition of -CH_2_COOH groups [20], which may also contribute to the intensity increase in the 1412 and 1324 cm^−1^ bands in the P+E film. This process increases the number of -CH_2_ groups and could explain the observed intensity increase, which suggests a more intense carboxymethylation process in the P+E film, likely influenced by the HPP treatment.

## 4. Conclusions

The combination of high pressure processing (HPP) and cellulase, followed by a carboxymethylation step of the eucalyptus pulp, allowed the production of cellulose films with several changed properties. More specifically, the combination of both treatments resulted in more smooth and transparent films, with higher moisture contents, tensile strength (TS) and elongation at break (EAB), water vapor permeability (WVP), and crystallinity indexes (CrI). On the other hand, the same combination resulted in a lower contact angle (on the bottom side) and a reduction in the thermal resistance of the cellulose films. Therefore, this study demonstrates that HPP and enzymatic pretreatments can be used to obtain carboxymethylated cellulose films with distinct properties. The next stages of this research should include optimization of the pressure, treatment time, and enzyme dosage, together with determination of the carboxymethylation yield, degree of substitution, carboxyl-group content, and molecular weight of the resulting cellulose derivatives. Further characterization of fiber morphology, fibrillation, surface roughness, glass transition temperature, and changes in color and transparency during storage would also support a more comprehensive understanding of the observed effects. Finally, application-oriented studies should assess and optimize water-vapor and gas-barrier performance, mechanical stability under relevant storage conditions, biodegradability, migration and food-contact safety, or biological performance, depending on the intended use of the films.

## Figures and Tables

**Table 1:** Molecular weight (g/mol) and composition (%) of the bleached kraft pulp.

	*E. globulus*
Molecular weight, g/mol	6648
Neutral Sugar Composition, %	
Glucose	82.9
Xylose	16.2
Rhamnose	0.3
Arabinose	0.1
Mannose	0.2
Galactose	0.3
Cellulose, % o.d. pulp	82.3
Hemicellulose, % o.d. pulp	17.0
Lignin, % o.d. pulp	0.33
Acetone extractives, % o.d. pulp	0.37

**Table 2:** Color parameters and transparency of the cellulose films (mean ± standard deviation). Different letters above the values (a, b, c, d) denote statistically significant differences (*p* < 0.05).

Sample	Color Parameters			Transmittance (%)
	L* (D65)	a* (D65)	b* (D65)	
Cnt	96.63 ± 0.13 ^bc^	−0.31 ± 0.02 ^a^	2.46 ± 0.05 ^a^	27.73 ± 3.33 ^a^
P	96.57 ± 0.28 ^ab^	−0.17 ± 0.03 ^b^	2.43 ± 0.02 ^a^	19.74 ± 2.79 ^b^
E	96.33 ± 0.25 ^a^	−0.27 ± 0.01 ^c^	3.14 ± 0.33 ^b^	15.61 ± 2.68 ^c^
P+E	96.83 ± 0.09 ^c^	−0.35 ± 0.03 ^d^	1.90 ± 0.32 ^c^	22.81 ± 2.98 ^a^

L* represents the lightness (black = 0 and white = 100), a* represents the greenness/redness (green = −a* and red = +a*) and b* represents the blueness/yellowness (blue = −b* and yellow = +b*) [39].

**Table 3:** Absorption bands of cellulose films and the groups they correspond to.

Absorption Bands	Groups	References
3350 cm^−1^	Stretching of the O-H groups	[49]
2924 cm^−1^	Stretching of the C-H groups	[49]
2362 cm^−1^	The absorption band of the atmospheric carbon dioxide that is present in the room where the FTIR analysis was performed	[50]
1586 cm^−1^	Presence of the COO^−^ groups	[49]
1412 and 1324 cm^−1^	Stretching in the plane and C-H stretching in symmetry	[49]
1054 cm^−1^	Stretching of the CH-O-CH_2_ groups	[51]
590 cm^−1^	Ring stretching and ring deformation of the α-D-(1-4) and α-D-(1-6) linkages	[52]

## Data Availability

The raw data supporting the conclusions of this article will be made available by the authors on request.

## Supplementary Materials

The following supporting information can be downloaded at: https://www.mdpi.com/article/10.3390/ma19163552/s1, Video S1: Cellulose films video.

## Abbreviations

The following abbreviations are used in this manuscript:

ANOVA	Analysis of variance
ATR	Attenuated total reflectance
CA	Contact angle
CAS	Chemical Abstracts Service registry number
CMC	Carboxymethylated cellulose/carboxymethyl cellulose
Cnt	Control batch/film
CrI	Crystallinity index
E	Enzymatically treated batch/film
EAB	Elongation at break
FTIR	Fourier-transform infrared spectroscopy
HPP	High-pressure processing
NS 280430	Endocellulase enzyme solution/product code
PA/PE	Polyamide/polyethylene
P	HPP-treated batch/film
P+E	HPP + enzymatically treated batch/film
RH	Relative humidity
SEM	Scanning electron microscopy
STA	Simultaneous thermal analyzer
TG	Thermogravimetric; used in “TG curves”
TGA	Thermogravimetric analysis
TS	Tensile strength
WT	Wettability
WRV	Water retention value
WVP	Water vapor permeability
XRD	X-ray diffraction
YM	Young’s modulus
